# Body composition as a novel biomarker of recurrence risk in patients with triple-negative breast cancer

**DOI:** 10.21203/rs.3.rs-5437121/v1

**Published:** 2024-12-17

**Authors:** Jill B. De Vis, Cong Wang, Kirsten V. Nguyen, Lili Sun, Brigitte Jia, Alexander D. Sherry, Mason N. Alford-Holloway, Meredith L. Balbach, Tatsuki Koyama, A. Bapsi Chakravarthy, Marjan Rafat

**Affiliations:** Vanderbilt University Medical Center; Vanderbilt Epidemiology Center; Vanderbilt University School of Medicine; Vanderbilt University Medical Center; Vanderbilt University School of Medicine; MD Anderson Cancer Center; Vanderbilt University Medical Center; Vanderbilt University Medical Center; Vanderbilt University Medical Center; Vanderbilt-Ingram Cancer Center; Vanderbilt University

**Keywords:** Prognostic markers, visceral-to-subcutaneous adiposity ratio, triple-negative breast cancer

## Abstract

**Background and Hypothesis:**

Triple-negative breast cancer (TNBC) patients are at increased risk for recurrence compared to other subtypes of breast cancer. Previous evidence showed that adiposity may contribute to worsened cancer control. Current measures of obesity, such as body-mass index (BMI), are poor surrogates of adiposity, while visceral-to-subcutaneous adiposity ratio (VSR), which can be measured from routine computed tomography (CT) imaging, is a direct adiposity measure. We hypothesized that VSR is a stronger predictor of recurrence compared with BMI in patients with TNBC.

**Materials and Methods:**

This study includes 162 women with stage I-III TNBC who completed standard of care therapy. Measures of body composition, including VSR, visceral adiposity (VA), and subcutaneous adiposity (SA), were estimated using a semi-automated quantitative imaging tool on CT images of the abdomen at the level of L2-L3. Anthropometric measures included BMI and waist circumference and were obtained from CT images. Associations of adiposity measures and recurrence risk were assessed using Fine and Gray competing risk models with death as a competing risk and age at diagnosis and clinical disease stage as covariates.

**Results:**

During a median follow-up time of 3.6 years, 55 patients had recurrence. The median BMI at baseline was 30.2 [Quartiles: 26.3–35.2]. Body composition was not associated with overall or locoregional recurrence. VSR was significantly associated with an increased risk of distant recurrence, with a subdistribution hazard ratio of 4.25 (95% CI: 1.06–17.02), p = 0.041. By contrast, BMI was not associated with any recurrence risk.

**Conclusion:**

Consistent with our hypothesis, VSR was associated with a significant risk of distant recurrence and therefore may be a prognostic biomarker. Future directions include interventions targeting VSR reduction among patients with TNBC and VSR-directed therapy modulation.

## Introduction

Breast cancer (BC) has a heterogeneous course that can largely be attributed to differences in clinical, histological, and molecular characteristics. About 15% of BC patients have triple-negative disease^1^, characterized by the absence of estrogen receptor, progesterone receptor and human epidermal growth factor receptor 2. Triple-negative breast cancer (TNBC) has a more aggressive course with increased risk of early recurrence, including distant recurrence^2^ as well as locoregional recurrence^3–5^, despite systemic treatment, surgery, and radiation. Currently, risk factors for recurrence of TNBC remain understudied. Therefore, TNBC would benefit from the identification of biomarkers for risk stratification and modulation.

Obesity has been identified as a risk factor for both hormone receptor positive as well as TNBC occurrence ^6^, recurrence^6,7^, and BC-related mortality^8^. Higher levels of circulating estrogens^9^, insulin resistance^10,11^, increased oxidative stress^12,13^, chronic inflammation^14–16^ and changes in adipocytokines^16,17^ have been attributed to these risks. Generally, anthropometric measures such as body mass index (BMI) or waist circumference that simultaneously capture visceral and subcutaneous adiposity (VA and SA) are used as biomarkers in epidemiologic studies investigating the effect of obesity on treatment outcomes. Whereas estrogen production, which is the main driver of recurrence risk in hormone receptor positive BC, is driven by both VA and SA, inflammation may be a more important factor in TNBC, which is largely caused by VA and has an inverse relation with SA^18^. Therefore, anthropometric measures may not accurately capture TNBC occurrence and outcome risk. Indeed, studies evaluating anthropometric measures as biomarkers in TNBC are conflicting with some studies reporting positive associations between obesity and TNBC occurrence^19^ and TNBC outcome^7,20,21^, while others have found negative associations^22,23^. A study that evaluated both central obesity and BMI as a biomarker for TNBC occurrence found central obesity to be a valid biomarker, while BMI was not^23,^ suggesting that VA may be a more accurate biomarker in TNBC patients.

The goal of this study was twofold: (1) to elucidate whether obesity is a predictive biomarker for TNBC recurrence, and (2) to identify measures of obesity that most reliably detect an increased risk of TNBC recurrence. We hypothesized that an elevated visceral-to-subcutaneous adiposity ratio (VSR), a measure of body composition, is associated with an increased risk of TNBC recurrence. This hypothesis was tested by examining the relationship between TNBC recurrence and anthropometric measures of obesity, BMI or waist circumference as compared to body composition measures, including VA, SA and VSR, to determine more accurate measures that can better predict patient outcomes.

## Methods

### Study subjects

Imaging and clinical data was collected from patients who had consented to enroll on an IRB approved Breast Tissue Repository (BRE03103, https://clinicaltrials.gov/study/NCT00899301). The inclusion criteria for our specific research question were women, at least 18 years of age, with histologically confirmed, invasive, stage I-III TNBC^24^, treated with standard-of-care treatment. Inclusion criteria also included patients for whom radiation was a component of management as both preclinical^5^ as well as retrospective studies^4^ have shown that radiation can increase the potential for inflammatory-associated recurrence risk. CT-simulation scans were used to determine measures of body composition through calculating VA, SA, and VSR.

### Data collection

Baseline characteristics including race, gender, age at diagnosis, and date of last follow-up or date of death were collected through electronic medical records (EMR) review. BC specifics including histology, hormone receptor status, human epidermal growth factor receptor 2 (HER2) status, pathologic stage, histologic grade, proliferative rate and lymphovascular invasion (LVI) were gathered. Radiation data included radiation dose to the breast or chest wall and regional lymph nodes, as well as dose to the tumor bed or scar and fractionation regimen. Recurrence was assessed as first site of recurrence being either locoregional with or without distant disease versus distant disease only. Time to recurrence and time to last follow-up were calculated as time from date of diagnosis to date of recurrence and date of last follow up or death, respectively.

### Obesity assessment

**Figure 1:**
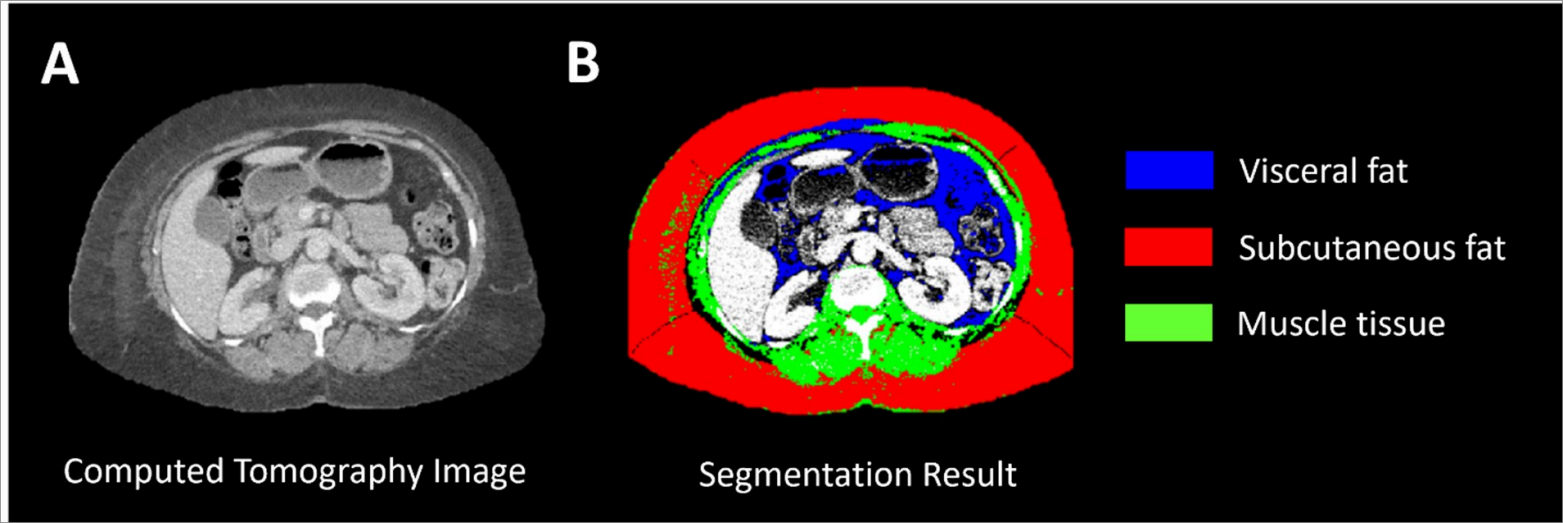
Representative computed tomography (CT) image to assess body composition. (**A**) CT image at the level of intervertebral disc lumbar vertebrae L2-L3. (**B**) Visual representation of the segmentation results with visceral fat in blue, subcutaneous fat in red, and muscle tissue in green.

Body Mass Index (BMI) was retrieved from the EMR, and waist circumference and body composition were evaluated using abdominal CT images, if available. All measurements were made at the time of initiating RT. A semi-automatic open-source MATLAB-based (MathWorks Inc., Natick, Massachusetts) segmentation tool was used to retrieve quantitative measures of waist circumference, VA, SA, and muscle tissue (Fig. 1) from the CT images. In brief, the tool allows for selection of a Digital Imaging and Communications in Medicine (DICOM) image of interest after which the body circumference is detected, and the intra-abdominal cavity is delineated semi-automatically using active contouring with boundary detection. Subcutaneous fat, muscle and visceral fat are then detected using fuzzy c-means clustering, boundary detection and Hounsfield Units thresholds. Results of the tool were validated by the developers through comparison with manual measurements, Aquarius (TeraRecon, Inc., Durham, NC, USA) and ImageJ (National Institutes of Health, Bethesda, MD, USA), with good performance (intraclass correlation coefficients ranging from 0.854 to 0.996)^25^. The above method does not allow for whole abdominal quantitative analysis but allows for single-level analysis which expanded our data collection from patients with early-stage TNBC disease who do not typically get staging CT scans. We focused on the intervertebral disc of lumbar vertebrae L2-L3, which has been shown to correlate best with total intra-abdominal fat^26,27^. To reduce noise in measurements, we processed three adjacent imaging slices and averaged the obtained measurements. Then, the visceral-to-subcutaneous fat ratio (VSR = VA/SA) was calculated to reflect the direct and inverse relation of VA and SA, respectively^18^.

### Statistical analysis

Patient baseline BMI, waist circumference and body composition characteristics (SA, VA, and VSR) were summarized using the median and quartiles for continuous variables or frequency and proportion for categorical variables. Pearson correlation was computed to estimate the association between two continuous variables. Comparisons between subjects with and without recurrence (for locoregional, distant and all recurrences) were conducted using chi-squared test for categorical variables and a linear-model analysis-of-variance (ANOVA) test for continuous variables. The Fine and Gray competing risk models^28^ were fitted to analyze time-to-event data, where the primary event of interest was recurrence (any, locoregional, and/or distant) with mortality treated as a competing risk. For graphical presentation of cumulative incidence curves, BMI, waist circumference, SA, VA, and VSR were categorized to high and low using the respective median values as the cut point. In each model, age at diagnosis and stage were included as covariates. The cumulative incidence functions were estimated for each type of event, and subdistribution hazard ratios were calculated to assess the effect of covariates on the risk of the primary event. All statistical analyses were performed using R version 4.3. The competing risk analyses were performed using the cmprsk package^29^. Two-sided P < 0.05 was considered statistically significant.

## Results

### Baseline characteristics

One hundred sixty-two women were included in this study (Table 1). Median age at the time of diagnosis was 54 [Quartile: 47–62] years old. Most lesions were invasive mammary carcinoma (98%), there were 2 metaplastic carcinomas (1%), and 2 subjects presented with inflammatory carcinoma (1%). Thirty percent of patients presented with stage I disease, 48% with stage II, and 22% of patients had stage III disease.

### Subject outcomes

During a median follow-up time of 7.1 [IQR 3.6–12.2] years, 56 (35%) patients developed recurrence; 31 patients (55%) presented with locoregional recurrence with or without distant recurrence at time of first recurrence, and 24 (43%) had distant recurrence only. Forty-four (27%) subjects died and, of these subjects, 35 (80%) were known to have disease recurrence. Median time to any recurrence was 1.9 [Quartiles: 1.5–3.0] years, with a median of 1.7 [Quartiles: 1.3–2.5] years for local recurrence and 2.1 [Quartiles: 1.8–3.7] years for distant recurrence.

Continuous variables are summarized with median followed by quartiles in parenthesis.

### Anthropometric and body composition measurements

**Figure 2:**
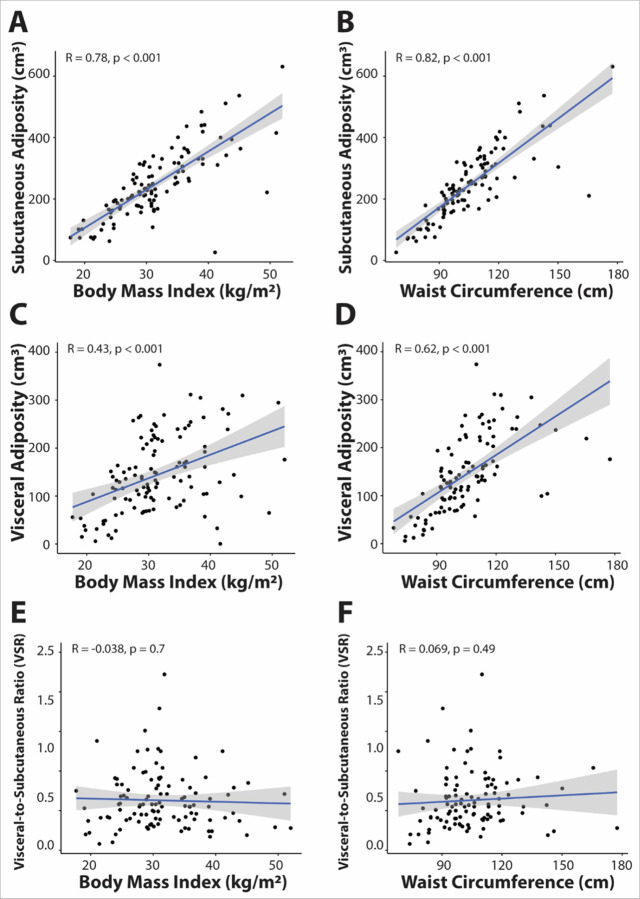
Correlation between body mass index, waist circumference, and body composition measurements (subcutaneous adiposity (SA), visceral adiposity (VA) and visceral-to-subcutaneous adiposity ratio (VSR). Scatter plots show SA (**A-B**), VA (**C-D**), and VSR (**E-F**) versus BMI and waist circumference with a simple regression line (blue) and 95% confidence interval in gray.

All subjects had BMI data available, while waist circumference and body composition measurements were available in 109 subjects (67%). The correlations between anthropometric measures (BMI and waist circumference) and body composition measures (VA, SA, and VSR) are shown in Fig. 2. SA strongly correlated with both BMI and waist circumference (r = 0.78 and 0.82, respectively, p < 0.001) (Fig. 2A, B) while the correlation of VA with BMI and waist circumference was moderate (r = 0.43 and 0.62, respectively) (Fig. 2C, D). In contrast, VSR was not correlated with BMI or waist circumference (r = −0.038 and 0.069, respectively) (Fig. 2E, F).

### Analysis of variables predictive of recurrence

BMI, waist circumference, VA, SA, and VSR were not significantly different on univariate analysis between patients with recurrence (locoregional, distant, or any recurrence) versus patients without recurrence (Table 2).

**Figure 3:**
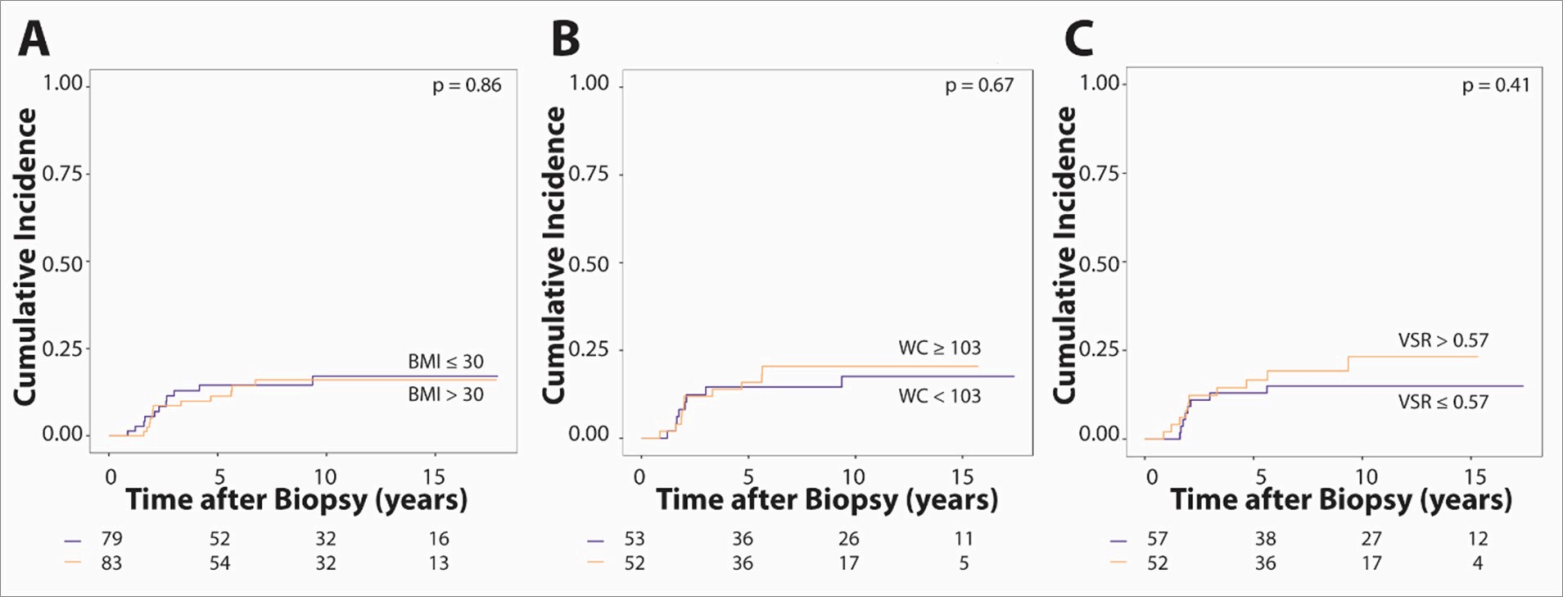
Cumulative incidence of distant recurrence stratified based on median anthropometric measures or VSR. Distant recurrence over time is shown for (**A**) body mass index (BMI; blue line, BMI ≤ 30 kg/m^2^; orange line, BMI > 30 kg/m^2^), (**B**) waist circumference (WC; blue line, WC < 103 cm; orange line, WC ≥ 103 cm), and (**C**) visceral-to-subcutaneous adiposity ratio (VSR; blue line, VSR ≤ 0.57; orange line, VSR > 0.57). A non-significant separation of the curves can be noted for VSR. At risk subjects are indicated along the x-axis.

Higher VSR was associated with a significantly increased risk of distant recurrence (Table 3), with a subdistribution hazard ratio of 4.25 (p = 0.04, 95% CI: 1.06–17.02). When dichotomized by median (0.57), VSR was not significantly associated with risk for distant recurrence (Fig. 3). We did not observe anthropometric or body composition measures including BMI to be associated with overall recurrence risk (Fig. 3, Supplemental Fig. S1 and S2).

## Discussion

In patients with TNBC, we evaluated obesity and adiposity measures as biomarkers for recurrence risk. Despite a largely obese cohort, we demonstrated that VSR, uniquely differs from anthropometric and other body composition measures, and may be a valuable biomarker for recurrence risk in TNBC patients. We demonstrated that anthropometric measures (i.e. BMI and waist circumference) poorly correlate with VSR, and VSR may have potential prognostic ability to distinguish distant recurrence risk.

We confirmed previously published data assessing the relationship between body composition assessment and anthropometric measures. A meta-analysis by Mouchti *et al.* found BMI and waist circumference to be strongly correlated with Magnetic Resonance Imaging-derived SA (r = 0.83–0.85) while the correlation with VA was less strong (r = 0.76–0.79), which is consistent with our data that shows a strong correlation between SA and anthropometric measures (r = 0.78 and 0.28 for BMI and waist circumference, respectively) and a moderate correlation with VA between BMI and waist circumference (r = 0.43 and 0.62, respectively)^30^. Work from Kaess *et al.* assessing VSR in the Framingham Heart Study cohort demonstrated a weak, positive correlation of VSR with BMI and waist circumference in women (r = 0.06 and 0.10, respectively)^31^. Our data also found that VSR is not correlated with BMI (r = −0.038) or waist circumference (r = 0.069), highlighting the validity of this body composition marker over anthropometric measures. Our data build on earlier reports that demonstrated increased risk of TNBC with elevated visceral adiposity^19^, and increased risk of cancer progression in BC patients with high visceral fat^32^, highlighting the importance of body composition assessments as biomarkers for disease recurrence through their association with transcriptome profiles^33^, and reinforcing the association of visceral or central obesity with cancer risk due to hyperinsulinemia^34,35^, chronic low-grade inflammation^33^ and oxidative stress^36^. Our study was not able to reproduce the effect of VA on tumor recurrence, likely due to our small sample size and largely obese cohort. However, our data suggest that VSR, quantifying visceral versus subcutaneous adiposity, may be a more sensitive biomarker for disease recurrence. This is consistent with data evaluating obesity measures and their association with cardiometabolic risk factors, which has also demonstrated that VSR is more highly correlated with cardiometabolic risk factors than BMI and VA^31^. The increased sensitivity of VSR for both cancer recurrence risk and cardiovascular disease risk may be explained by the ability of VSR to simultaneously capture the positive association of visceral adipose tissue, as well as the inverse relation of subcutaneous adipose tissue with insulin resistance^18^. Accordingly, visceral fat is thought to cause hepatic insulin resistance via release of non-esterified fatty acids and inflammatory mediators in the portal venous system^37,38^ in contrast to subcutaneous fat^39^. Insulin resistance or hyperinsulinemia is a known important risk factor for both cardiovascular disease^40^ and TNBC risk^41^. Hyperinsulinemia drives cancer risk by (1) promoting cellular proliferation and neoangiogenesis, (2) overproducing reactive oxygen species which introduce mutagenesis and carcinogenesis, and (3) inhibiting apoptosis^42,43^. Interestingly, a prior study demonstrated a correlation between VSR and vascular endothelial growth factor (VEGF), a potent angiogenic factor, which supports the above hypothesis^44^.

Our study has some limitations. First, our findings are restricted by a small sample size and skewed towards an obese Caucasian cohort related to the geographical location of our institution^45^. Validation of these findings with a larger sample size in a different geographic area with a wider range of body composition and a more diverse population is important. Data processing of a larger cohort may be facilitated by implementing artificial intelligence to assess body composition^46^. Next, patients included in our study were diagnosed and received treatment between 2004 and 2021, which means that many of our patients with locally advanced disease did not receive current standard-of-care treatment with immune checkpoint inhibition (ICI). Some reports suggest that the association between obesity and cancer progression may be altered in patients receiving ICI, and future studies should evaluate whether this is the case for TNBC patients^47–49^. Lastly, prospective studies are needed to assess the effect of lifestyle modifications on body composition and disease recurrence risk. Thiazolinediones, a class of insulin sensitizers, have been shown to expand SA while reducing VA,^50^ resulting in improved insulin sensitivity^51^ and thus may be a potential protective agent. Despite these limitations, we have shown that evaluating VSR may be an improved method of quantifying obesity compared to the current anthropometric standard and may predict recurrence risk in TNBC patients. While we present correlative evidence, future studies with increased power and more diverse patient populations may lead to using VSR not only as a prognostic factor but also to effect change using FDA-approved semaglutide products to meaningfully reduce patient body weight^52^.

## Conclusions

The data in this study demonstrate that the ratio of visceral-to-subcutaneous adipose tissue is a potential prognostic factor for risk of distant recurrence risk in TNBC patients. Future work will validate this finding in a larger cohort and may focus on therapeutic strategies to improve this ratio by decreasing contribution of visceral fat to total body fat.

## Supplementary Material

Supplement 1

## Figures and Tables

**Table 1:** Baseline patient characteristics

	N (%)
Age	Median 54, Quartiles (47–62)
Race	
Caucasian	119 (73)
Black	35 (22)
Hispanic	4 (2)
Asian	1 (1)
Unknown	3 (2)
Laterality	
Left	79 (49)
Right	83 (51)
Stage	
I	46 (30)
II	74 (48)
III	34 (22)
Grade	
High	127 (79)
Intermediate	29 (18)
Low	5 (3)
Lymphovascular invasion	
Yes	28 (18)
No	102 (65)
Unknown	26 (17)
Body Mass Index (kg/m^2^)	30 (26–35)
Waist Circumference (cm)	102 (93–113)
Subcutaneous Adiposity (cm^3^)	232 (177–313)
Visceral Adiposity (cm^3^)	133 (91–203)
Visceral-to-Subcutaneous Adiposity Ratio	0.56 (0.38–0.81)

**Table 2:** Anthropometric values (BMI, waist circumference) and body composition measurements (VA, SA, VSR) in patients with and without recurrence.

	No recurrence	Locoregional recurrence	Distant recurrence	Any recurrence
Body Mass Index (kg/m^2^)	30 [26–35]	31 [28–37]	30 [26–32]	31 [27–35]
Waist Circumference (cm)	102 [93–114]	103 [96–113]	105 [96–112]	104 [95–113]
Subcutaneous Adiposity (cm^3^)	229 [179–311]	262 [183–316]	235 [167–293]	246 [178–314]
Visceral Adiposity (cm^3^)	130 [79–178]	138 [91–238]	157 [92–212]	140 [91–222]
Visceral-to-Subcutaneous Adiposity Ratio	0.5 [0.4–0.8]	0.6 [0.5–0.8]	0.6 [0.4–0.9]	0.6 [0.4–0.8]

**Table 3:** Subdistribution Hazard Ratio and 95% confidence interval of competing risk models.

	Locoregional recurrence	Distant recurrence	Any recurrence
Body Mass Index (kg/m^2^)	1.01 (0.97–1.06) p = 0.55	0.98 (0.92–1.04) p = 0.49	0.99 (0.96–1.03) p = 0.85
Waist Circumference (cm)	1.00 (0.98–1.02) p = 0.98	0.99 (0.98–1.01) p = 0.60	0.99 (0.98–1.01) p = 0.71
Subcutaneous Adiposity (cm^3^)	1.00 (1.00–1.00) P = 0.74	0.99 (0.99–1.00) p = 0.36	0.99 (0.99–1.00) p = 0.35
Visceral Adiposity (cm^3^)	1.00 (0.99–1.01) P = 0.96	1.00 (0.99–1.01) p = 0.23	1.00 (0.99–1.01) p = 0.63
Visceral-to-Subcutaneous Adiposity Ratio	0.91 (0.26–3.21) P = 0.88	4.25 (1.06–17.02) **p = 0.04**	1.53 (0.64–3.62) p = 0.34
