# Supplementary material for: Body composition as a novel biomarker of recurrence risk in patients with triple-negative breast cancer
Source: Res Sq. 2024 Dec 17:rs.3.rs-5437121. Preprint. [Version 1] doi: 10.21203/rs.3.rs-5437121/v1 (PMC11702791; doi:10.21203/rs.3.rs-5437121/v1)
Supplement: Supplement 1 [file NIHPPRS5437121v1-supplement-1.pdf]

Supplementary Files

This is a list of supplementary files associated with this preprint. Click to download.

- BodyCompositionSIfinal.docx
